# Psychopathological Predictors of Suicidal Intentions in ICU Patients and a Multimodal Preventive Psychotherapeutic Framework

**DOI:** 10.1192/j.eurpsy.2026.10693

**Published:** 2026-07-31

**Authors:** O. Davydenko, B. Mykhaylov

**Affiliations:** Shupyk National Healthcare University of Ukraine, Kyiv, Ukraine

## Abstract

**Introduction:**

Suicidal behaviour is a major public health issue, yet its occurrence in somatic intensive care units (ICUs) remains understudied. ICU patients often face extreme physical stress, psychological trauma, and social isolation, which can trigger suicidal ideation, even in the absence of prior mental illness.

The combination of chronic somatic disease, perioperative stress, and impaired cognitive-emotional regulation increases vulnerability to suicidal thoughts and behaviours. Despite this, targeted preventive strategies in ICU settings are limited, and psychiatric symptoms are frequently overlooked or misattributed to physical illness.

This study aims to synthesise current knowledge on suicidal tendencies among somatic ICU patients, identify key psychopathological predictors, and propose a multimodal psychotherapeutic prevention program tailored to this high-risk group.

**Objectives:**

To review current knowledge on suicidal intentions and tendencies among patients in intensive care units (ICUs) of somatic hospitals, to identify key psychopathological predictors and to propose a multimodal preventive psychotherapeutic program.

**Methods:**

A literature review of international and Ukrainian sources on suicidal behaviour in ICU patients, combined with clinical observations, was conducted. Factors such as psychiatric comorbidity, chronic somatic diseases, perioperative psychological stress, and cognitive-affective predictors were analyzed to develop structured prevention strategies.

**Results:**

Key predictors of suicidal behavior among ICU patients included bradypsychia, delusional self-blame, hypomimia, attention disorders, and depressive affect, even in individuals without prior mental health disorders. A multimodal psychotherapy program was developed, integrating cognitive-behavioral therapy, EMDR techniques, and autogenic training, implemented in three stages: sedative-adaptive, therapeutic-corrective, and prophylactic-consolidating. The program emphasizes interdisciplinary collaboration, phased intervention, and combining biological and psychological approaches to reduce suicide risk and improve psychosocial adaptation.

**Conclusions:**

Understanding specific suicidal predictors in ICU patients allows for prompt preventive interventions. The proposed multimodal program may reduce suicidal mortality, enhance rehabilitation outcomes, and facilitate patients’ return to active social life. Future research should focus on protocol validation and integrating preventive strategies into routine ICU care.

**Disclosure of Interest:**

None Declared

